# Seasonality of Hypoplastic Left Heart Syndrome and Single Ventricle Heart in Poland in the Context of Air Pollution

**DOI:** 10.3390/jcm10153207

**Published:** 2021-07-21

**Authors:** Iwona Strzelecka, Małgorzata Biedrzycka, Filip Franciszek Karuga, Bartosz Szmyd, Katarzyna Batarowicz, Maria Respondek-Liberska

**Affiliations:** 1 Department for Diagnoses and Prevention, Medical University of Lodz, 93-338 Łódź, Poland; iwona.strzelecka@umed.lodz.pl (I.S.); batarowicz.katarzyna@gmail.com (K.B.); maria.respondek-liberska@uni.lodz.pl (M.R.-L.); 2 Student’s Scientific Association Prenatal Cardiology, Medical University of Lodz, 93-338 Łódź, Poland; filipfranciszek439@gmail.com; 3 Department of Pediatrics, Oncology, and Hematology, Medical University of Lodz, 91-738 Łódź, Poland; bartoszmyd@gmail.com; 4 Department of Prenatal Cardiology, Polish Mother’s Memorial Hospital, 93-338 Łódź, Poland

**Keywords:** hypoplastic left heart syndrome, single ventricle, congenital heart defect, prenatal cardiology, seasonality, air pollution

## Abstract

Hypoplastic left heart syndrome (HLHS) and single ventricle (SV) remain a significant cause of cardiac deaths occurring in the first week of life. Their pathogenesis and seasonal frequency are still unknown. Therefore, we attempt to look at the genesis of the HLHS and SV in the context of territorial distribution as well as seasonality. A total of 193 fetuses diagnosed with HLHS and 92 with SV were selected. The frequency was analyzed depending on the year, calendar month, quarter and season (fall-winter vs. spring-summer). The spatial distribution of HLHS and SV in Poland was analyzed. We observed a statistically significant overrepresentation of HLHS formation frequency in March: 27 (14.00%) in comparison to a monthly median of 15 (IQR: 13.75–16.25; *p* = 0.039), as well as a significantly higher frequency of HLHS in 2007−2009: 65 cases (33.68%) in comparison to the annual mean of 13.79 ± 6.36 (*p* < 0.001). We noted a higher frequency of SV among parous with the last menstrual period reported in the fall/winter season of 58 vs. 34 in the spring/summer season (*p* = 0.014). The performed analysis also revealed significant SV overrepresentation in 2008: 11 cases (12.00%) in comparison to the annual mean of 6.57 ± 2.71 (*p* = 0.016). Every single case of HLHS was observed when the concentration of benzo(a)pyrene and/or PM10 exceeded the acceptable/target level. Our research indicates that both the season and the level of pollution are significant factors affecting the health of parous women and their offspring. The reason why HLHS and SV develop more frequently at certain times of the year remains unclear, therefore research on this topic should be continued, as well as on the effects of PM10 and benzo(a)pyrene exposure.

## 1. Introduction

Hypoplastic left heart syndrome (HLHS) belongs to the group of congenital heart defects (CHD) described as univentricular hearts [1]. Most broadly, HLHS includes any number of lesions with a dominant right ventricle (RV) and systemic outflow obstruction that are not amenable to two-ventricle repair [2]. In each case, underdevelopment of the left-sided structures of the heart may involve aortic valve atresia, atresia or stenosis of the mitral valve, hypoplasia of the ascending aorta and aortic arch. [3].

Usually HLHS and single ventricle heart (SV) share a common pathway of postnatal treatment (multistage repair), however these defects have different genesis [4,5,6,7]. Unfortunately, SV is not an unequivocal term as it may be considered in anatomical and functional way. Rao in his current, comprehensive review stated that functional SV is primarily used to describe any congenital heart defect with one functioning ventricle (double-inlet left ventricle, single ventricle, common ventricle, and univentricular atrio-ventricular connection) [8]. Moreover, he noted that other lesions, such as HLHS, tricuspid atresia, unbalanced AV septal defect, mitral atresia with normal aortic root, and heterotaxy syndromes with one functioning ventricle may now be added to this group [8]. The functional SV may have a different prognosis than anatomical SV. Therefore, in this study, the heart was considered SV when there was no evidence of an interventricular septum on echocardiography performed in the fetal or neonatal life. We included hearts with two atria and two atrioventricular valves or common atrium with one atrioventricular valve, single ventricle (regardless of the type of trabeculation), two outflows in criss-cross relation, or parallel relation, in some cases coexisting disproportion between two arteries. Fetuses from this group were qualified for palliation cardiac surgery after birth. Our goal was to make a distinction between HLHS and anatomical SV and to focus on the possible different environmental influences during embryogenesis, despite similar cardiac surgery pathways in postnatal life.

**Figure 1:**
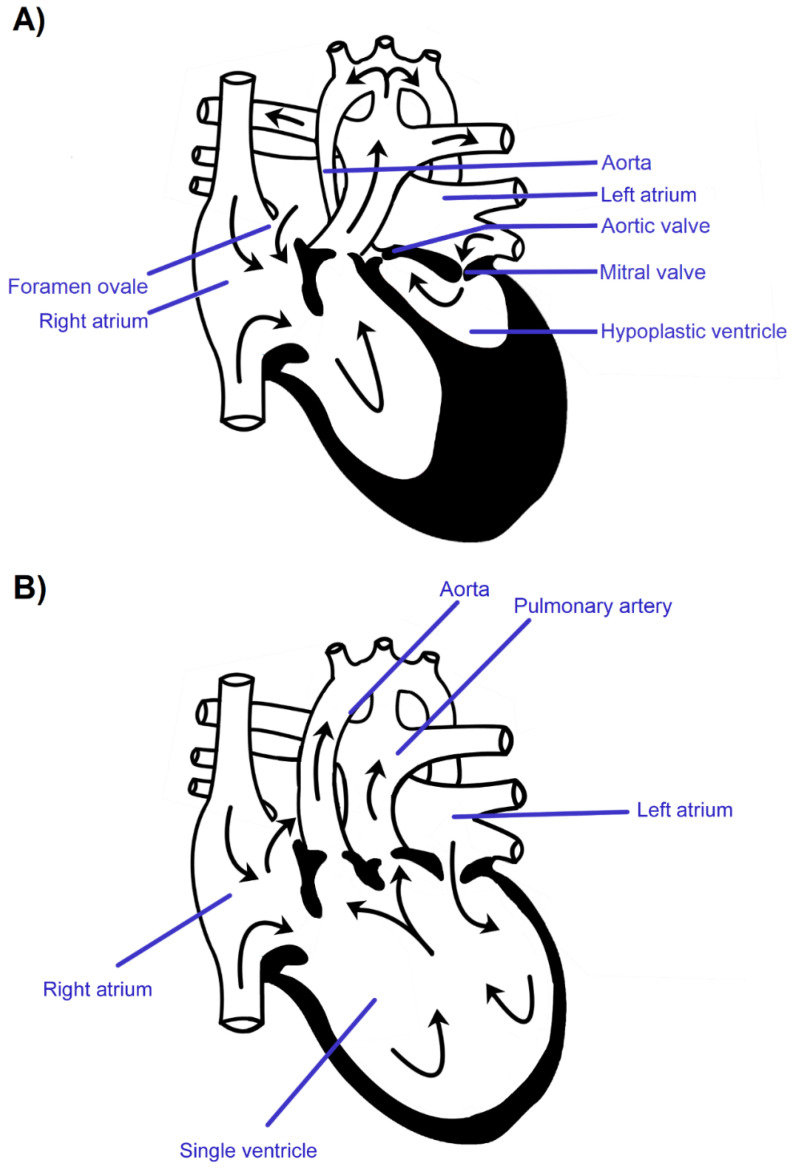
The schematic figure of fetal hypoplastic left heart syndrome (**A**) and anatomical single ventricle heart (**B**).

**Figure 2:**
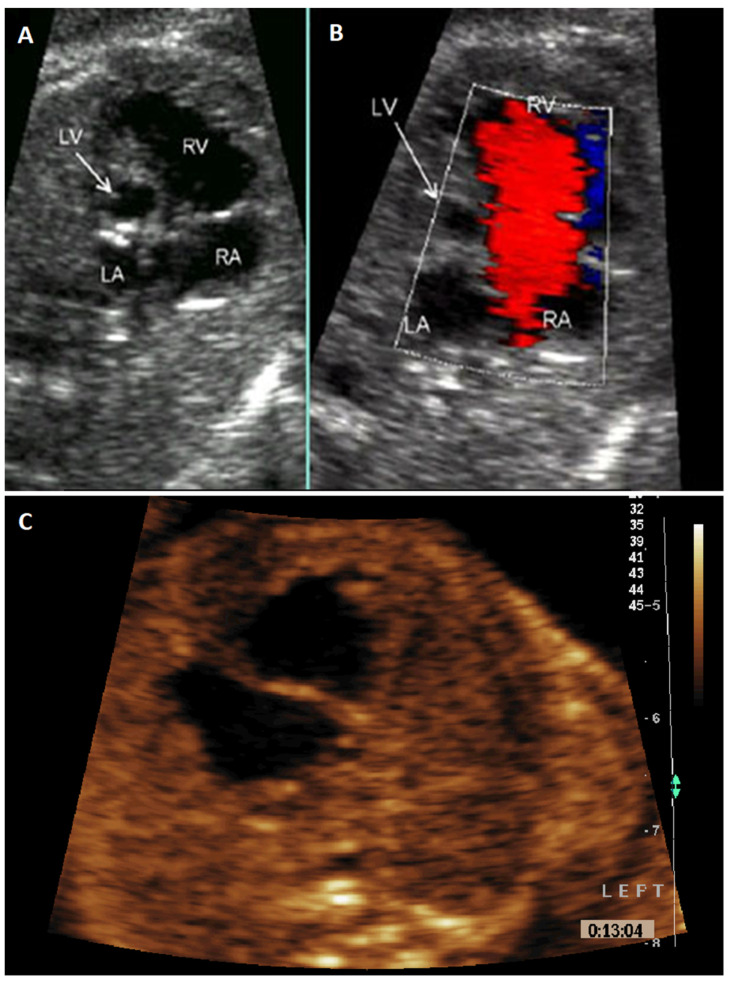
(**A**) Hypoplastic left heart syndrome fetal echocardiogram in a four-chamber view with noticeably reduced dimensions of the left ventricle compared to the right ventricle. (**B**) The same heart presented in color Doppler echocardiography. The embryogenesis of this heart is usually at 6–8th week after conception or even later on. (**C**) Single fetal echocardiogram. This heart in terms of embryology was earlier developed comparing with hypoplastic left heart syndrome, usually 4–5th week after conception. Legend: LA—left atrium, LV—left ventricle; RA—right atrium, RV—right ventricle.

Anatomical SV forms in week 4 of embryogenesis and only one ventricle is formed in the heart at a time. Fetuses and newborns with this malformation have a better chance of survival [9,10,11]. HLHS is usually formed in the 6th week of pregnancy or even later on. After the initially correct division of the heart into four chambers, the development of the left ventricle is inhibited for various reasons, with the accompanying changes in the development of the adjacent structures of the left heart and the vessels carrying blood from the left ventricle [12,13,14]. The schematic figures and corresponding fetal echocardiogram images presenting HLHS and anatomical SV are shown in Figure 1 and Figure 2.

According to the European Surveillance of Congenital Anomalies (EUROCAT), CHDs account for nearly one-third of major congenital anomalies diagnosed prenatally or in infancy in Europe [15]. Among those 1.4–3.8% correspond to HLHS. Despite the relatively low incidence, it is responsible for 23% of cardiac deaths occurring in the first week of life [16]. Thus, it is crucial to find all possible relationships between HLHS and risk factors for the occurrence of the defect in the population, which in most cases are unknown [17,18,19,20].

In this study, we attempt to look at the genesis of the HLHS and SV in the context of territorial distribution in relation to air pollutant levels: benzo(a)pyrene (B(a)P) and particulate matter 10 μm or less in diameter (PM10), as well as seasonality.

## 2. Materials and Methods

### 2.1. Study Group Selection

Out of 17,174 echocardiographic examinations performed on 11,690 pregnant women in our single reference center in the middle of Poland for prenatal cardiology in 2006–2019, 2115 were diagnosed with a fetal heart defect. To exclude the multiple inclusion of the same case in the statistics, the first-time confirmation was selected for the analysis. A total of 193 fetuses diagnosed with an isolated heart defect in the form of HLHS and 92 with SV were selected. The date of the maternal last menstrual period (LMP) was used as a time frame for fetal heart defects formation: LMP + 6 weeks for SV formation and LMP + 8 weeks for HLHS formation. Only singleton pregnancies were analyzed. Fetuses with confirmed genetic abnormalities (e.g., abnormal karyotypes), as well as cases where dysmorphia, especially craniofacial one, were excluded from further analysis. Pregnancies after IVF were also excluded, as well as fetuses from the abroad population (or immigrant population). None of the families had a history of CHD. None of the mothers had an infection or smoked during pregnancy. In the SV group, only one mother had Hashimoto treated with levothyroxine. In the HLHS group, 3 mothers had hypothyroidism and 2 had gestational diabetes. The other women were neither chronically ill nor taking medications. All cases of prenatally diagnosed HLHS or SV were confirmed after birth by neonatal echo or by an autopsy in our Institution. Cases with lost follow-up were not counted. The places of patients’ residency were analyzed from our unit database. Before the fetal exam every patient was asked for a written consent agreement suggesting the use of their data for future research.

### 2.2. Measurement Tools

The frequency of CHDs was analyzed depending on the year, as well as the calendar month, quarter and season (fall-winter vs. spring-summer). Moreover, the spatial distribution of HLHS and SV in Poland was analyzed with division into 46 administration zones. These are 12 urban agglomerations with more than 250,000 inhabitants, 18 cities with more than 100,000 inhabitants, and 16 zones that are areas of voivodships not included in these cities or agglomerations. Therefore, data comes from urban measuring stations, as well as from stations located outside the cities. There are usually several or a dozen measuring stations in each zone. Official government data available on the website of the Chief Inspectorate for Environmental Protection was used to assess the possible relationship between HLHS and SV pathogenesis and two air pollutant levels in assessed zones [21]. To compare the level of air pollution, only data for 2010–2019 were taken into account. This is due to the fact that earlier official government data were not uniform and 46 administration zones were not established yet.

### 2.3. Statistical Analysis

Chi-square test, chi-square test with Yates correction or Fisher’s exact test were performed for nominal data depending on the smallest group number. The results for nominal data are presented as n (% of total). Results were further validated using logistic regression analysis (a forward stepwise model). The normality of continuous data distribution was assessed by Shapiro−Wilk test (*p* > 0.05–normal distribution). These data were reported as mean ± SD—normal distribution or median with interquartile range (IQR) in other cases. A *p* < 0.05 was considered statistically significant. Statistical analysis was performed using Statistica 13.1 (StatSoft, Tulsa, OK, USA).

## 3. Results

### 3.1. Variability Over Time

**Figure 3:**
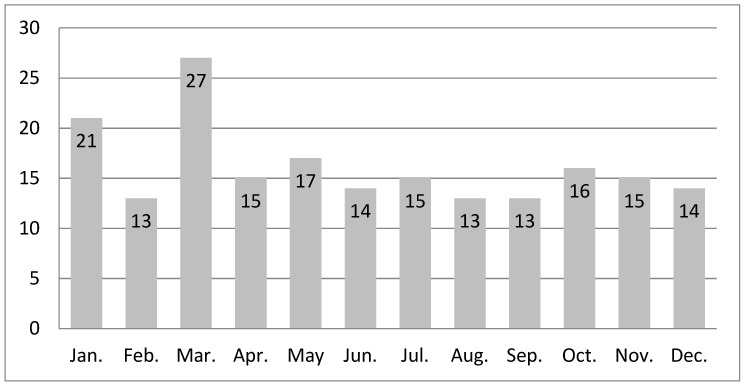
Cumulative numbers of hypoplastic left heart syndrome cases formed in consecutive months in years 2006–2019 (*n* = 193, data from our single unit). Overrepresentation of frequency of hypoplastic left heart syndrome formation in March: 27 (14.00%) in comparison to a monthly median of 15.

We observed a statistically significant overrepresentation of frequency of HLHS formation in March: 27 (14.00%) in comparison to a monthly median of 15 (IQR: 13.75–16.25; *p* = 0.039, χ^2^ test). Cumulative numbers of HLHS formed in consecutive months in years 2006–2019 are presented in Figure 3. Cumulative numbers and percentages of HLHS and SV in consecutive months in years 2006–2019 are presented in Table 1. 

We observed a visible peak of HLHS cases in the years 2007– 2009. The use of the χ^2^ test confirmed a statistically significant increase in this period compared to the remaining observation time: 65 cases (33.68%) in comparison to the annual mean of 13.79 ± 6.36 (*p* < 0.001). Further validation with the usage of logistic regression analysis (a forward stepwise model) showed that year significantly modulates the risk of HLHS (OR = 1.164, 95% CI: 1.095–1.236, *p* < 0.001; see Table 2A).

**Figure 4:**
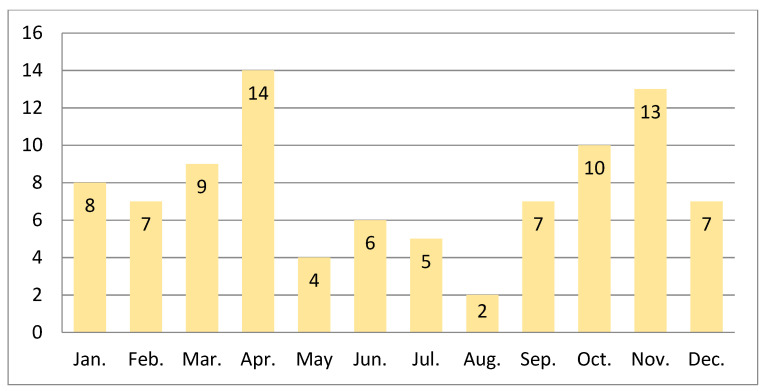
Cumulative numbers of single ventricle cases formed in consecutive months in years 2006– 2019 (*n* = 92, data from our single unit). Higher frequency among parous with the last menstrual period in fall/winter season of 58 vs. 34 in spring/summer season (*p* = 0.014, chi-square).

Concerning SVs, we noted their higher frequency among parous with the last menstrual period reported in the fall/winter season 58 vs. 34 in the spring/summer season (*p* = 0.014, chi-square). Cumulative numbers of SV formed in consecutive months in years 2006–2019 are presented in Figure 4. Moreover, performed analysis revealed also significant SV overrepresentation in 2008: 11 cases (12.00%) in comparison to the annual mean of 6.57 ± 2.71 (*p* = 0.016, χ^2^ test). These results were further validated by logistic regression analysis (a forward stepwise model), which showed that seasons significantly modulate the SV risk (OR = 1.739, 95% CI: 1.132–2.670, *p* = 0.011; see Table 2B). 

### 3.2. Territorial Distribution

**Figure 5:**
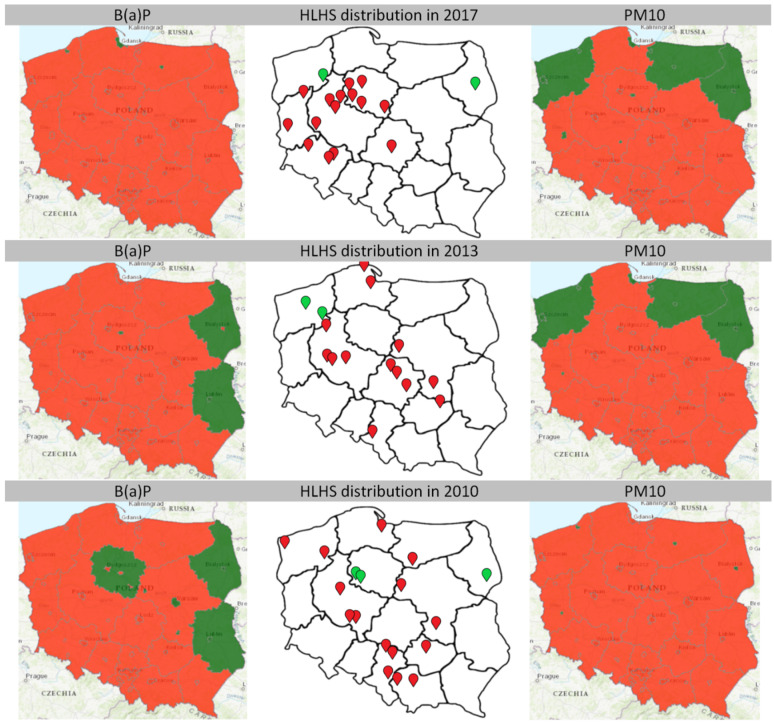
Levels of air pollution (B(a)P on the left and PM10 on the right) and territorial distribution of hypoplastic left heart syndrome cases in years 2017, 2013 and 2010. The red area corresponds to exceeding the permissible levels, in the green area the concentration of the measured factor is normal. In the middle, the distribution of all hypoplastic left heart syndrome cases in a given year is marked—the red dots correspond to the cases in the areas where both factors were above the norm and the green ones—where the concentration of one of the factors was normal. B(a)P—benzo(a)pyrene, HLHS—hypoplastic left heart syndrome.

The data provided by the Chief Inspectorate for Environmental Protection in the years 2010−2019 revealed that every single case of HLHS (112 cases) was observed when the concentration of B(a)P) and/or PM10 exceeded the acceptable/target level (C category). Interestingly, only 3 HLHS cases were observed when the B(a)P concentration was within the norms (all in 2010). Moreover, only 4 HLHS cases were noticed during normal concentrations of PM10 (2013 and 2017). The territorial distribution of HLHS cases, as well as maps presenting concentration of B(a)P and PM10 in years 2010, 2013 and 2017 are presented in Figure 5. In total, 93.75% of HLHS cases occurred when in a given area concentration of at least one air pollutant exceeded the norms. 

**Figure 6:**
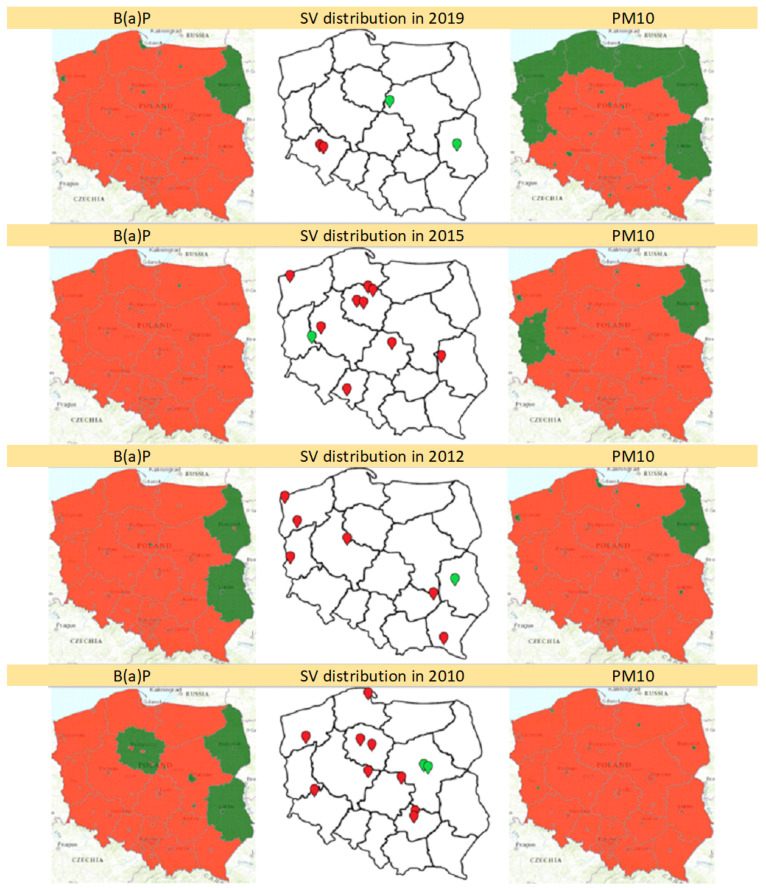
Levels of air pollution (benzo(a)pyrene on the left and PM10 on the right) and territorial distribution of single ventricle cases in years 2019, 2015, 2012 and 2010. The red area corresponds to exceeding the permissible levels, in the green area the concentration of the measured factor is normal. In the middle, the distribution of all single ventricle cases in a given year is marked—the red dots correspond to the cases in the areas where both factors were above the norm and the green ones—where the concentration of at least one of the factors was normal. B(a)P—benzo(a)pyrene, SV—single ventricle.

In the years 2010–2019, 62 cases of SV were observed. Only 2 cases were noticed during normal concentrations of both B(a)P and PM10 in a given area (2012, 2019). Among the others, only 2 SV cases were observed during normal concentrations of B(a)P (both in 2010), and 2 during normal concentrations of PM10 (2015, 2019). In total, 56 (90.32%) SV cases occurred when in a given area concentration of both air pollutants exceeded the norms. Sixty (96.77%) SV cases occurred when the concentration of at least one air pollutant was exceeded. The territorial distribution of SV cases, as well as maps presenting concentration of B(a)P and PM10 in years 2010, 2012, 2015 and 2019 are presented in Figure 6.

## 4. Discussion

### 4.1. Variability Over Time

The most significant finding of our study is that throughout the year, not all of the analyzed “similar” CHDs occur with the same frequency. For HLHS, the maximum frequency of its formation falls in March, and SV seems to be most frequent in parous with the last menstrual period reported in fall/winter (October–March).

So far, the available sources are ambiguous as for the seasonal variability of HLHS. Some report that their research did not show any statistically significant differences between the compared periods [22,23]. An example may be a study by Sokołowski et al., who concluded that no clear seasonal pattern was evident and the values were not differing significantly between the four seasons, but he made no difference between HLHS and SV [22].

Other researchers present data supporting the existence of such variability [24,25]. Dong et al. based on data from 2006−2017, concluded that in the Chinese population among patients with congenital heart disease there exists a statistically significant seasonal trend in the monthly birth rate [24]. The lowest numbers were recorded in April and the highest in October. Based on that and Naegele’s Rule, they concluded that maternal exposure to potentially harmful factors probably occurred in the period from January to March. These results are consistent with those obtained in our study. Moreover, it is worth noting that their research was conducted in a time frame similar to ours. Dai et al. found that for various CHD, the peak birth months were September and October [26]. Thus, fertilization and heart organogenesis fell during the winter period. 

On the other hand, considering HLHS births exclusively, Eghtesady et al. showed that significant seasonal differences occurred in each year of the study, with peaks in summer months (monthly maximum in June) and troughs in winter months (January) [25]. 

### 4.2. Potential Effect of Air Pollution on HLHS Formation

When it comes to the HLHS, the reasons for its formation are not entirely clear. Although prior studies suggest that HLHS has a complex genetic inheritance, its etiology remains largely unknown [27]. HLHS is a CHD that is especially well documented to accompany various genetic disorders [28]. Some of the genetic syndromes in which HLHS is found include Turner syndrome, trisomy 13, trisomy 18, partial trisomy 9, Smith−Lemli−Opitz, Holt−Oram [13,29]. Jacobsen syndrome is a rare chromosomal disorder caused by deletions in distal 11q and was determined to have a wide spectrum of congenital heart defects, including unprecedented high frequency of HLHS which occurs in 5–10% of all patients with 11q deletion [29].

HLHS has been linked with several mutations in mouse models: *Sap130*, *Pcdha9* [28,30]. In humans, heterozygous mutations in NKX2.5 and GJA1 have been reported in a small number of cases [13]. Results of the study conducted by Tomita-Mitchell et al. reveal that a significant percentage of HLHS patients have rare and damaging *MYH6* variants (including a novel, missense, in-frame deletion, premature stop, de novo, and compound heterozygous variants) and are predictive of poor clinical outcomes in the form of reduced transplant-free survival [31].

On the other hand, the number of studies showing the influence of nongenetic factors is growing [32,33,34,35]. Feng et al. report that maternal factors such as low educational level, smoking, binge drinking, medicine consumption (especially analgesics and antibiotics), and influenza during pregnancy are associated with an increased risk of their offspring developing CHD [36]. As for other factors, heat exposure during early pregnancy [37], maternal vitamin D deficiency, exposure to air pollutants in pregnancy, and even structural or vascular placental abnormalities seem to be relevant [24,35,37,38,39].

Cronk et al. in their study sought to corroborate the clinical impression of excess HLHS cases in eastern Wisconsin. The calculated HLHS birth prevalence was equal to 3.7 per 10,000 births, which was greater than the estimated population risk of 2.79. The rates were highest in the most urban and industrialized areas, such as the southeast region. Because of the geographic clustering of high rates, the authors concluded that environmental factors may be associated with elevated HLHS occurrence in this part of the state. Interestingly, in this study, no increased incidence for TGA or tetralogy of Fallot (ToF) was found in those areas [40].

In another study, Cronk et al. examined models of the geographic distribution of HLHS, TGA and ToF over a 10-year period in Eastern Wisconsin. This time, a significant south-to-north spatial gradient for HLHS, TOF, and combined CHDs, but not TGA was identified. They concluded that geographic variation may be an indicator of risk factors for birth defects, and therefore further studies controlling, among others, linkage to potential environmental exposures (such as agrichemicals or groundwater contamination) are needed to identify risk factors that may underlay this geographic gradient [41].

Among air pollutants, nitrogen dioxide, sulfur dioxide and PM2,5 have the strongest evidence for interfering with heart embryology [42,43,44,45]. These can lead to the formation of such heart diseases as CoA ToF, pulmonary valve stenosis or ventricular septal defect [42,43,44,45].

Continuous maternal exposure to concentrations of PM10 exceeding the norm is associated with increased risk for multiple congenital heart defects [46], for instance ToF [43,44,45,46,47]. Patent ductus arteriosus or atrial septal defects are not congenital heart defects from a prenatal cardiology point of view. Since they are physiologically shunts during fetal life, only their permanence after the birth is considered a defect. Mixing prenatal and postnatal defects could be a source of bias. Stingone et al. found a positive association between HLHS and exposure to PM2.5, but not between HLHS and PM10 or NO_2_ [32]. So far, researchers did not show any correlation specifically between HLHS and PM10 [43,45,47,48].

Benzo(a)pyrene is a representative of polycyclic aromatic hydrocarbons and a known human carcinogen. Furthermore, it can also affect female reproductive health and pass through the placental barrier [49]. B(a)P can cause enhanced cell apoptosis and inhibited migration, leading to trophoblast dysfunction and pregnancy loss [50]. Its metabolites can also cause choriocarcinoma, intrauterine growth restriction and eclampsia [49]. A relation between B(a)P and fetal heart was reported by Więckowska et al. in their research analyzing the occurrence of heart tumors in fetuses in Poland over 22 years. According to this study, cardiac tumors were recorded mainly in regions of high concentration of B(a)P and other polycyclic aromatic hydrocarbons (i.e., benzene) [51].

To the best of our knowledge, the presented research is the first study to show a relationship between concentration levels of B(a)P and PM10 and the occurrence of HLHS and SV. We draw this conclusion on the basis that every case of HLHS originated in areas with a high air concentration of at least one from two parameters: B(a)P or PM10. In general, the concentration levels of both pollutants were in category C—this means that the concentration was above the acceptable (for PM10) or target (for B(a)P) level. Under the Environmental Protection Law, in class C zones, specific activities are required to achieve appropriate levels of permissible substances in the air [21].

According to the annual reports of the Chief Inspectorate for Environmental Protection, exceeding the target levels of B(a)P and PM10 is mainly associated with its high levels in the winter. This, in return, results from high emissions (especially from the sources of the so-called low emissions related to the heating of buildings) and worse dispersion conditions in the cold season [21]. Interestingly, in our study, SV seems to be most frequent in parous with the last menstrual period reported in fall/winter (October–March), thus in the period with the highest level of air pollution. Poland has relatively well-developed prenatal diagnostics and a high degree of air pollution, therefore it is a good place to assess this type of relationship.

In the years 2010–2019, almost all SV cases (96.77%) occurred when, in the mother’s place of residence, concentrations of at least one investigated air pollutant exceeded the norms. Only two cases (3.2%) appeared when both—B(a)P and PM10—were below an acceptable level.

As for HLHS, only seven cases (6.25%) appeared when in the given area, one of the indicators did not exceed the norm: three cases with B(a)P at an acceptable concentration level (all in 2010) and four with PM10 (two in 2013 and two in 2017). These cases may be due to imperfect measurements. Since 2010, the number of zones in which the levels of pollution are measured has been constant and equals 46. However, the number of measuring stations has significantly increased, and thus the accuracy of the measurements. In 2010, data came from 27 measuring stations for B(a)P and 66 for PM10, in 2019 it was 157 and 313 stations, respectively. Therefore, the highest number of cases that occurred when one parameter was normal (three cases) are in 2010, and in 10 subsequent years, there are only two cases in years 2013 and 2017. Additionally, as can be seen in Figure 5, some of these seven cases of HLHS occurred in zones with normal concentrations of B(a)P or PM10, however, very close to the border with the red zone. This may mean that the actual exposure of women to air pollution in these places could be greater than the reading in the given zone indicates.

## 5. Strengths and Limitations

A strong point of our study is that the impact of nonenvironmental factors has been minimized. Fetuses with abnormal karyotypes or craniofacial dysmorphia were excluded. There was no family history of CHD in any of the cases. We also ruled out the influence of factors such as maternal smoking or infections during pregnancy. Except for a few cases, the mothers did not suffer from chronic diseases or take medication.

Another strong point is that the data were obtained by experienced prenatal cardiologists in tertiary-level cardiology centers [52,53]. The prenatal detection rate for HLHS is usually high: depending on the population, they exceed 85% for univentricular defects as the abnormal four-chamber cardiac view can be seen during the screening exam [54,55]. However, some cases presented in the literature suggest that a normal four-chamber view at mid-gestation does not necessarily rule out heart defects in the third trimester of pregnancy [56]. Thus, in prenatal diagnostics, the level of experience of the individuals performing the ultrasonography, as well as the standards of equipment, play a key role.

The main limitation of our study is that we compared the occurrence of HLHS in given zones with the annual level of pollution in that area because data in such a form is provided by the Chief Environmental Inspectorate. When it comes to pollution levels in Poland, data obtained from such a source seems to be the most accurate (as measurements and devices must meet specified standards) and allows for a reliable comparison of data between consecutive years. The downside, however, is that the annual scope and the reference to entire zones do not give us confidence that these data perfectly correspond to the exposure of a given pregnant woman at a given time. Another barrier to creating smaller subdivision zones (following the principle: one measuring station = one zone) is the fact that the number of stations in each zone was not constant in the years included in our study as it increased almost every year. This fact makes comparing such unequal zones in the following years extremely difficult and could lead to error. For better data reliability, it would be ideal to compare the contamination levels by sampling areas that were closest to the woman’s place of residence in the suspected time of defect formation. 

## 6. Conclusions

Serious health implications are associated with HLHS and SV diagnosis, so apart from the possibility of early detection, it is especially important to determine and reduce and impact of modifiable risk factors. Our research indicates that both the season and the level of pollution are significant factors affecting the health of parous women and their offspring. Therefore, further studies are needed on the effects of PM10 and B(a)P exposure, as well as the seasonal variability of HLHS and SV. Furthermore, our study shows that there is a difference in etiology and epidemiology between SV and HLHS. In some studies, they are considered as one group, which may shed new light on previous works and their interpretations.

## Figures and Tables

**Table 1:** Cumulative numbers and percentages of hypoplastic left heart syndrome and single ventricle cases in consecutive months in years 2006–2019.

	Jan.	Feb.	Mar.	Apr.	May	Jun.	Jul.	Aug.	Sep.	Oct.	Nov.	Dec.	Total
HLHS	21	13	27	15	17	14	15	13	13	16	15	14	193
%	10.9%	6.7%	14.0%	7.8%	8.8%	7.3%	7.8%	6.7%	6.7%	8.3%	7.8%	7.3%	100.0%
SV	8	7	9	14	4	6	5	2	7	10	13	7	92
%	8.7%	7.6%	9.8%	15.2%	4.3%	6.5%	5.4%	2.2%	7.6%	10.9%	14.1%	7.6%	100.0%

**Table 2:** The logistic regression model (a forward stepwise model) testing the parameter affecting HLHS (A) and SV (B) frequency.

**A. Parameters affecting HLHS frequency**			
	OR	95% CI	*p* value
Intercept	0.000	0.000–0.000	<0.001
Year	1.163	1.095–1.236	<0.001
**B. Parameters affecting SV frequency**			
	OR	95% CI	*p* value
Intercept	0.006	0.004–0.008	<0.001
Year	1.739	1.132–2.670	0.011

## Data Availability

Data are available upon request, please contact Małgorzata Biedrzycka (malgorzata.biedrzycka@stud.umed.lodz.pl).
